# Genetic Regulation of Sinoatrial Node Development and Pacemaker Program in the Venous Pole

**DOI:** 10.3390/jcdd2040282

**Published:** 2015-11-30

**Authors:** Wenduo Ye, Yingnan Song, Zhen Huang, Yanding Zhang, Yiping Chen

**Affiliations:** 1 Department of Cell and Molecular Biology, Tulane University, New Orleans, LA 70118, USA; E-Mails: ysong4@tulane.edu (Y.S.); zhuang3@tulane.edu (Z.H.); 2 Southern Center for Biomedical Research, Fujian Normal University, Fuzhou 350108, China; E-Mail: ydzhang@fjnu.edu.cn; 3 Fujian Key Laboratory of Developmental and Neural Biology, College of Life Science, Fujian Normal University, Fuzhou 350108, China

**Keywords:** pacemaker development, venous pole, atrial fibrillation, SAN, pulmonary vein

## Abstract

The definitive sinoatrial node (SAN), the primary pacemaker of the mammalian heart, develops from part of pro-pacemaking embryonic venous pole that expresses both Hcn4 and the transcriptional factor Shox2. It is noted that ectopic pacemaking activities originated from the myocardial sleeves of the pulmonary vein and systemic venous return, both derived from the *Shox2*^+^ pro-pacemaking cells in the venous pole, cause atrial fibrillation. However, the developmental link between the pacemaker properties in the embryonic venous pole cells and the SAN remains largely uncharacterized. Furthermore, the genetic program for the development of heterogeneous populations of the SAN is also under-appreciated. Here, we review the literature for a better understanding of the heterogeneous development of the SAN in relation to that of the sinus venosus myocardium and pulmonary vein myocardium. We also attempt to revisit genetic models pertinent to the development of pacemaker activities in the perspective of a Shox2-Nkx2-5 epistatic antagonism. Finally, we describe recent efforts in deciphering the regulatory networks for pacemaker development by genome-wide approaches.

## 1. Introduction

The orchestrated contraction of the four-chambered mammalian heart is highly coordinated by the cardiac conduction system (CCS). The matured CCS of mammals contains the sinoatrial node (SAN), atrioventricular node (AVN), AV bundle and ventricle conduction networks [1]. The primary pacemaker, the SAN, is embedded in the junction between the right superior vena cava (RSVC) and the right atrium (RA). The SAN cells maintain highest automaticity and function to provide the primary impulse for each coordinated contraction. Dysregulation of SAN development and homeostasis causes sick sinus syndrome (SSS) [2].

### 1.1. The SAN Can Be Divided into Subdomains by Anatomical and Genetic Criteria

Initially noted as “a small condensed area of tissue located just where the cava sinks into the auricle” more than 100 years ago [3], the SAN is now recognized as a complex functional unit and the subdomains within the SAN have been defined both anatomically and genetically. Anatomically, a “head” region of the SAN can be identified as a histologically discrete structure that extends superiorly and wraps around the RSVC. The SAN also extends inferiorly toward the inferior vena cava (IVC) to form a “tail” structure [4,5,6,7].

**Figure 1:**
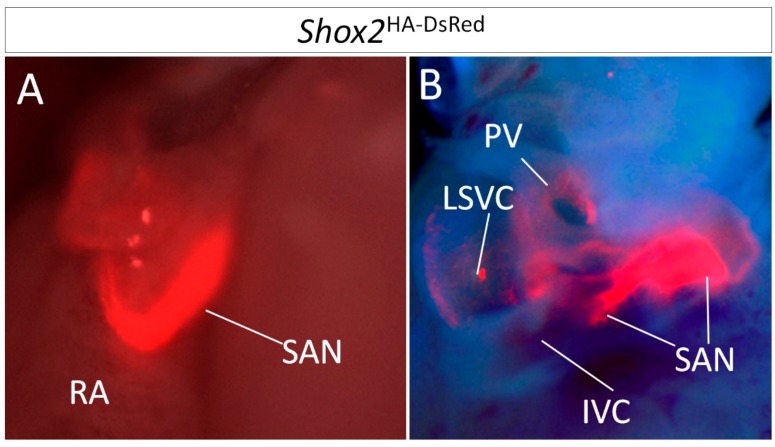
Visualization of *Shox2* expression in the SAN by *Shox2*^HA-DsRed^ allele. (**A**) Frontal view of *Shox2* expression in the SAN of a mouse carrying a *Shox2*^HA-DsRed^ allele at Postnatal Day 8 (P8), as revealed by DsRed expression. (**B**) Dorsal view of *Shox2* expression in the same mouse. PV, pulmonary vein; RA, right atrium; IVC, inferior vena cava; SAN, Sinoatrial node; LSVC, left superior vena cava.

Genetically, in the mouse, the SAN can be divided into a *Tbx18*^+^/*Nkx2-5*^−^ head domain and a *Tbx18*^−^/*Nkx2-5*^+^ tail/peripheral domain. In addition, the entire SAN can be identified by the expression of *Shox2*, as shown in Figure 1, as well as *Tbx3*, *Isl1*, and *Hcn4* [8,9,10,11]. A number of reporters/markers were also used to identify the developing SAN, such as the *CCS-LacZ* transgene [12] and the surface marker HNK-1 [13], though their specific SAN localization was not indicated. While the distinct functions of the SAN head and SAN tail/peripheral domain have not been well characterized, a recent study indicates that the *Nkx2-5*^+^ SAN tail/peripheral domain is essential for normal SAN function [9].

### 1.2. Lineage Development of the SAN in Relation to Other Venous Pole Structures

**Figure 2:**
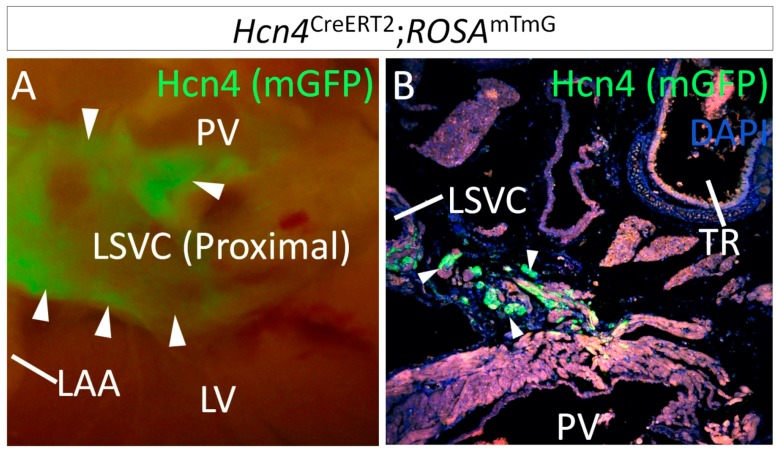
*Hcn4* expression in the proximal junction of the PV and LSVC myocardium of an adult mice. (**A**) Dorsal view of EGFP expression (arrowheads) in an adult *Hcn4*^CreERT2^; *ROSA*^mTmG^ mouse seven days after tamoxifen induction. (**B**) *Hcn4* positive cells (arrowheads) in the junction between the PV and LSVC of the same mouse. LV, left ventricle; PV, pulmonary vein; TR, Trachea; LAA, left atrial appendage; LSVC, left superior vena cava.

During early development, after the formation of a linear heart tube through bilateral cardiogenic plate fusion (the first heart field, FHF), additional progenitor cells are continuously recruited from the surrounding mesenchyme to the outflow tract and the venous pole (inflow tract) where the SAN resides [14,15,16,17]. A morphologically distinct SAN structure can be identified as early as Embryonic Day 9.5 (E9.5) in the mouse. The initial function of the SAN was thought to start at around E12.5 [18] in the mouse, with typical SAN-like action potential (AP) configurations being identified at E14.5 [9,19]. Although the SAN is the dominant pacemaker in the venous pole, cells with pacemaking activities can be found not only in the SAN, but also in the myocardial sleeves of the systemic venous return and the pulmonary vein (PV) myocardium. It was proposed that these cells with pacemaking activities can act as ectopic pacemaker to trigger atrial fibrillation (A-Fib) [20,21], making the characterization of the lineage origin of these sites in relation to the SAN an interesting subject. Multiple lineages have been shown to contribute to the development of the venous pole based on marker expression and genetic fate mapping studies, such as the *Tbx18*^+^/*Nkx2-5*^−^ sinus venosus (SV) cells that develop into the myocardial sleeves of the systemic venous return [14,22], the posterior second heart field (SHF) cells mapped by *Mef2C-AHF-Cre* that develop into the primary atrial septum [16,23], and the cells labeled by *Hcn4Cre*^ERT2^ induced from E9.5 onward [24]. Based on the observation that *Tbx18*^+^ lineage also maps to the SAN, it was assumed that the SAN is derived mainly from the *Tbx18*^+^/*Nkx2-5*^−^ SV myocardial cells [14,22]. Interestingly, the lineage and genetic attribution of the myocardial sleeves around the PV have been under heavy debate regarding whether it is a SV-like lineage. Similar to the SV myocardium, the PV myocardial cells are *CCS-LacZ* positive and HNK-1 positive as well [25,26]. However, unlike the SV myocardium, the PV myocardium is derived from a *Tbx18*^−^/*Nkx2-5*^+^ lineage [27], arguing against the hypothesis that the PV myocardium and SV myocardium share a common origin. Our recent study showed that the PV myocardium, SV myocardium, as well as SAN are all derived from *Shox2*^+^ cells [9], offering a new perspective for understanding the lineage attribution of the PV myocardium. Interestingly, by using the expression of *Shox2* and *cTnT* as a marker for myocardial cells, a *Shox2*^+^/*cTnT*^+^/*Hcn4*^+^ myocardial continuation was clearly identified in the proximal junction of the left superior vena cava (LSVC) and the PV as early as E10.5, supporting a common origin of the SV and PV myocardium [9]. In adult mice, this group of cells remains positive for *Hcn4* (Figure 2), suggesting a potential etiology for A-fib if the counterpart of these cells is present in humans. Moreover, *Shox2* is expressed in the PV side of the left atrial-PV structural continuum [9], providing a molecular marker for distinguishing the PV derived structure from the atrial-PV confluence structural continuum. Thus, although some genes, such as *Tbx3* and *Isl1*, are the SAN specific markers, other genes that mark the SAN also serve as lineage markers for the venous pole such as *Tbx18* that labels the SV myocardium and *Shox2* that labels the SV myocardium along with the PV myocardium. Such correlation coincides with the knowledge that the SV and PV myocardial cells are prone to develop foci induced A-Fib [20,21], and that the *Shox2*^+^ SV and PV myocardial cells display pacemaker-like characteristics at early embryonic stages [9,20,21]. Given that pacemaker properties are shared among *Shox2*^+^ cells at early developmental stages regardless of the expression of *Tbx3* and *Isl1* that are predominantly expressed in the SAN [9,18], an interesting questions is raised: is the genetic program that controls pacemaker properties in the venous pole independent from the one that controls the development of the histologically discrete SAN?

## 2. Genetic Models for SAN Development

To further understand SAN development in relation to the SV and PV myocardium, we sought to revisit literature describing mouse models generated for studying SAN development. We categorized these models by whether or not mutations would cause phenotypes in both the venous pole and SAN.

### 2.1. Genes and Genetic Models for SAN Dysgenesis without Complications in the SV and PV Myocardium

**Figure 3:**
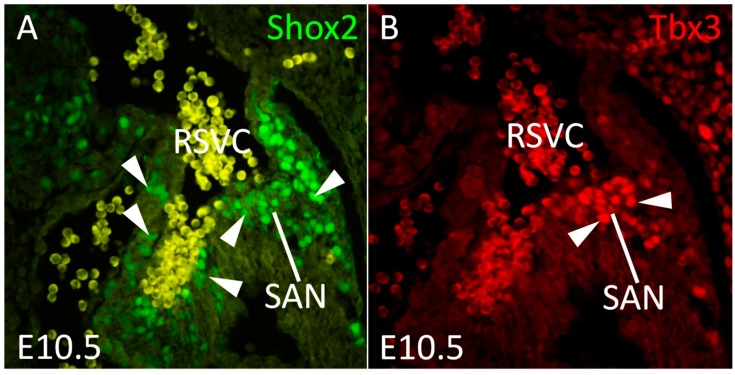
Expression of *Shox2* and *Tbx3* in the SAN at E10.5. Immunofluorescence shows the expression of *Shox2* (arrowheads) in the SAN and other tissues around the SAN (**A**) and the restricted expression of *Tbx3* (**B**) (arrowheads) in the SAN. SAN, sinoatrial node; RSVC, right superior vena cava.

*Tbx3*: *Tbx3*, encoding a T-Box transcription factor, has been well characterized for its essential role in embryonic development and postnatal function of the SAN and AVN. Null mutation or hypomorphism of *Tbx3* causes sick sinus syndrome and atrial-ventricular (A-V) conduction block [8,28,29,30,31,32]. Tbx3 acts predominantly as a molecular repressor on the expression of working myocardial specific genes in the SAN [1,33]. It has been proposed that Tbx3 functions to compete with Tbx5 for binding with Nkx2-5, thus repressing the activation of the target genes of *Nkx2-5* and *Tbx5* such as *Scn5α* (encoding Nav1.5) and *Gja5* (encoding Cx40) [1,33]. Tbx3 may also repress *Nkx2-5* expression directly as conditional inactivation of *Tbx3* elicits ectopic activation of *Nkx2-5* in the adult SAN [32]. Interestingly, although it has been established that *Hcn4* is a repressive target of *Nkx2-5* [9,34], null mutation of *Tbx3* does not cause downregulation of *Hcn4*, suggesting that *Tbx3* is only partially required for the maintenance of the SAN program. The fact that *Tbx3* is expressed in a manner more restricted than that of *Shox2* (Figure 3) in the SAN region at E10.5 when the dominant pacemaker activity is not yet confined to the SAN [18] suggests that the definitive SAN cells are already specified before the establishment of the dominant pacemaker activity and require a distinctive genetic program likely centering around *Tbx3*.

*Isl1*: *Isl1* encodes a LIM-domain homeodomain transcription factor. Although *Isl1-Cre* maps to the whole SHF derivatives of the mouse heart, *Isl1* is expressed later on only in a small subpopulation of cells in the heart including the SAN [35,36]. Recently, the essential function of *Isl1* in the development and function of the SAN was revealed by genetic and transcriptome studies in an *Isl1*^F/F^; *Hcn4*^ERT2Cre/+^ model in which the deletion of *Isl1* is induced at stages beyond E10.5 at which point the role of *Isl1* in maintaining the progenitor state of SHF cells becomes relatively limited and its deletion is less likely to complicate the analysis of *Isl1* function in the SAN [10,11]. As it was expected, inactivation of *Isl1* causes bradycardia associated with reduced size of the SAN and down-regulation of a number of SAN specific genes including *Hcn4*, *Shox2*, and *Tbx3* [10,11].

### 2.2. Gene and Genetic Models Affecting Both the SAN and Other Venous Pole Components

*Tbx18*: *Tbx18* is the first gene that was shown to be able to convert non-pacemaker cardiomyocytes into pacemaker-like cells when it was forcedly expressed [37,38]. *Tbx18* is expressed in the pro-epicardial organ (PEO), developing SV myocardium, and SAN head in the venous pole [39]. *Tbx18-Cre* could also map to part of the SAN tail/peripheral domain, but not to the PV myocardium [39]. Accordingly, inactivation of *Tbx18* causes dysgenesis of both the SAN head and the SV myocardium [39]. Interestingly, *Tbx18* is never expressed in the PV myocardium, arguing for a different lineage origin of the PV myocardium *versus* the SV myocardium. Although the SAN head undergoes severe dysgenesis in *Tbx18* mutants, a functional SAN tail/peripheral domain is retained, implying the existence of an unidentified mechanism that controls pacemaker development in the SAN tail/peripheral domain and possibly in the PV myocardium as well.

*Shox2: Shox2* belongs to the family of short stature homeobox genes and is expressed in the developing SV and PV myocardium, and the SAN in both mice and humans [9], as well as in a small subdomain of the dorsal mesenchymal protrusion [19]. Previous studies showed that *Shox2* is essential for SAN development partially by preventing *Nkx2-5* expression in the SAN head [40,41]. Interestingly, *Tbx3* is also essential for repressing the *Nkx2-5* expression in the SAN head [32], raising the possibility that *Shox2* and *Tbx3* repress *Nkx2-5* synergistically to ensure normal SAN development. This hypothesis is supported by the observation that mice carrying compounded *Shox2* and *Tbx3* hypomorphic alleles have significant higher level of *Nkx2-5* in the SAN compared to the littermate controls [42]. Actually, *Shox2* and *Nkx2-5* are co-expressed extensively in the SAN tail/peripheral region that is also *Tbx3* positive and in a group of cells surrounding the SAN. In addition, *Shox2* and *Nkx2-5* are also co-expressed in the other venous pole components, such as the PV myocardium that is *Hcn4* positive in early embryonic stages and possesses pacemaker properties, suggesting an unidentified mechanism for *Shox2* to control pacemaker development [9]. It was proposed that the *Shox2-Bmp* pathway may function to regulate SAN development [19,43]. However, *Shox2-Cre* mediated site specific inactivation of either *Bmp4*, the major BMP ligand that is highly expressed in the SV and SAN myocardium, or *Smad4*, which is required for the execution of canonical BMP signaling, results in a normal functional SAN [44]. These observations indicate that although *Shox2* is required for the developmental expression of *Bmp4* in the venous pole, the *Shox2-Bmp4* pathway does not sufficiently account for the function of *Shox2* in SAN development.

*Nkx2-5*: Numerous studies have pinpointed to the importance of *Nkx2-5* in early cardiac development, maturation of cardiomyocytes, and A-V conduction axis. The role of *Nkx2-5* in the venous pole, however, was underappreciated, partially due to embryonic lethality of *Nkx2-5* null mice around E10.5, precluding functional analysis of *Nkx2-5* at later stage of venous pole morphogenesis. *Nkx2-5* is initially absent from the SV myocardium and the SAN head [9,20] but is acquired in the SV myocardium at around E14.5. The PV myocardium is *Nkx2-5*^+^ and expresses a relatively weak level of *Hcn4* expression compared to the SV myocardium [9,45]. In an *Nkx2-5* hypomorphism model in which the expression level of *Nkx2-5* is reduced to about 25% of wide type level [45,46], the PV myocardium acquires strong *Hcn4* expression and losses the expression of *Gja5* (*Cx40*) partially, mimicking the SV myocardial phenotype [45]. Furthermore, hypomorphism of *Nkx2-5* also results in an “invasion” of SAN phenotype to the surrounding atrial tissue, suggesting that the PV myocardium and the atrial tissues peripheral to the SAN are “default” to a pacemaker-like cell fate and such a “default” fate is suppressed by the presence of *Nkx2-5* [45]. It was reported recently that this pacemaker “default” fate is primed and sustained by the presence of *Shox2* [9]. Nevertheless, it is generally accepted that *Nkx2-5* exerts a repressive effect on pacemaker program by facilitating the maturation of cardiomyocytes in the developing venous pole. Such notion is further supported by the observation that venous pole specific deletion of *Nkx2-5* in the *Sln-Cre*; *Nkx2-5*^F/F^ mouse model results in an enlarged SAN [47].

*Pitx2*: Components of the four-chambered heart, including venous pole structures, undergo left-right asymmetric development at early stage. It has been demonstrated that *Pitx2*, encoding a homeodomain transcription factors, confers left sided patterning information [48,49,50]. It is noted that the myocardial sleeves of the LSVC and PV are positive for *Pitx2* and were considered “left sided” structures [51,52]. *Pitx2* deficiency leads to the emergence of right-sided structures including the SAN and venous valves (VV) in the left-side SV-atrial junction [18]. Moreover, it was shown that haploinsufficiency of *Pitx2* caused A-Fib and ectopic expression of SAN program including *Tbx3* and *Shox2* in the left-side of the heart [53,54]. Although the loss of either *Nkx2-5* or *Pitx2* results in the ectopic pacemaker phenotype, inactivation of *Pitx2* leads to an acquirement of the complete pacemaker program in the left SV-atrial junction, whereas haploinsufficiency of *Nkx2-5* causes only an upregulation of *Hcn4* in the *Pitx2*^+^ left sided structure, suggesting that these two genes repress pacemaker program through independently functional mechanisms [53,54]. Such hypothesis is further supported by an observation that in some *Pitx2*^−^ right sided structures such as left venous valve, haploinsufficiency of *Nkx2-5* elicits an activation of a relatively complete SAN program including *Tbx3* and *Hcn4* [42].

## 3. *Shox2-Nkx2-5* Antagonistic Mechanism in the Regulation of Pacemaker Properties

### 3.1. Shox2-Nkx2-5 Antagonistic Mechanism

To date, genetic, biochemical, and electrophysiological studies have suggested that pacemaker cells retain properties that are common for the early primitive cardiomyocytes such as poorly organized sarcomere and the expression of *Hcn4* that is partially responsible for the high automaticity [55]. Thus it is reasonable to assume that rather than taking a positive acquiring process for pacemaker phenotype, SAN development may undergo a preventive process from maturation towards working myocardial cell fate. Such assumption is well in line with the fact that many machineries required for the maintenance of pacemaker properties are also utilized for sustaining progenitor state of the anterior SHF cells. A good example is that *Isl1*, which is essential for maintaining the undifferentiated state of the SHF cells, is also crucial for SAN development [10,11]. Moreover, similar to its function in the differentiation of the anterior SHF cells, *Nkx2-5* also promotes cardiomyocyte maturation in the venous pole, and *Tbx3* was shown to act as a transcription repressor in the SAN for the working myocardial program that is enforced by *Nkx2-5* and *Tbx5* [28]. However, these transcriptional machineries do not account sufficiently for the pacemaking activities in the SV myocardium and PV myocardium where *Tbx3* and *Isl1* are not expressed. Given that the derivatives of the SV and PV myocardium are prone to acting as triggers for A-Fib [20], characterization of the molecular mechanism that enforces pacemaker properties in these sites would be important for developing gene based therapeutic approaches for A-fib.

Based on the fact that *Shox2* is co-expressed with *Hcn4* in the PV and SV myocardium where *Isl1* and *Tbx3* are not expressed, and that the *Shox2*^+^ PV myocardium cells display pacemaker-like properties, *Shox2* was believed to be a good candidate responsible for the potential pacemaker properties in the SV and PV myocardium independent of the SAN program genes *Tbx3* and *Isl1* [9]. Since hypomorphism of *Nkx2-5* causes augmentation of *Hcn4* expression in the *Nkx2-5*^+^/*Shox2*^+^ PV myocardium [9], and that the *Shox2*^+^/*Hcn4*^+^ SV myocardium is negative for *Nkx2-5*, it was proposed that *Nkx2-5* inhibits pacemaker properties that belong to a primitive cell phenotype, and such effect is counter-balanced by *Shox2*. By genetic approaches, it was established that a *Shox2-Nkx2-5* antagonistic mechanism controls pacemaker *versus* working myocardial cell phenotype in the PV myocardium, SV myocardium, and part of the SAN [9]. Consistent with the observation that hypomorphism of *Nkx2-5* results in an “invasion” of SAN phenotype to the surrounding atrial tissue [45], we have recently observed that the tissues being preferentially “invaded” by SAN phenotype are all primed by *Shox2* expression [42], suggesting the balance between *Shox2* and *Nkx2-5* transcription output is critically maintained to restrict the size of the SAN.

### 3.2. Heterogeneous Development Model of the SAN and the Essential Physiological Function of the Shox2^+^/Nkx2-5^+^ SAN Tail/Peripheral Domain

Similar to that in the PV myocardium, *Shox2* and *Nkx2-5* are also co-expressed in the developing peripheral SAN and function to regulate the development of this SAN domain by their antagonistic action [9]. The development of the SAN tail/peripheral domain appears to be independent of the SAN head, as the deletion of *Shox2* in the SAN peripheral domain by *Nkx2-5-Cre* (*Shox2*^Nkx2-5Cre^) led to virtual absence of the SAN tail/peripheral region but an unaffected SAN head [9]. In line with this notion is the fact that null mutation in *Tbx18* results in a severely hypoplastic SAN head but leaves the SAN tail/periphery unaffected [8]. The physiological importance of the peripheral region of the SAN was initially implied by *Scn5α* heterozygous mutants that exhibit sick sinus syndrome associated with SAN exit block [56]. *Scn5a* encodes a sodium channel protein (Nav1.5) and is expressed in the developing heart including the peripheral region of the SAN but not the SAN head. However, direct evidence supporting an essential role of the peripheral SAN in normal SAN function and S-A conduction was not available, primarily due to the lack of tools or genetic models to inactivate genes specifically in the peripheral SAN domain. In *Shox2*^Nkx2-5Cre^ model, a severe sick sinus syndrome was observed associated with the absence of the peripheral SAN [9], providing the first line of direct evidence for the functional importance of the peripheral SAN as an integrated part of the SAN. Furthermore, although previous studies have supported the notion that *Nkx2-5* plays an inhibitory role in general on pacemaker program in the venous pole, the conserved expression of *Nkx2-5* in the SAN tail/periphery in both mice and humans suggests an underappreciated unique role for *Nkx2-5* in SAN development and function. The fact that Nkx2-5 binds directly to a verified enhancer downstream of *Scn5α*, revealed by Nkx2-5 ChIP-Seq results [9,57,58], suggests that *Nkx2-5* regulates *Scn5α* expression directly in the peripheral SAN domain to confer a unique electrophysiological property to the SAN tail/periphery. This hypothesis is supported by our recent observation that mice bearing the deletion of *Nkx2-5* in the SAN peripheral domain also exhibited sick sinus syndrome [42]. We therefore propose a model that the *Shox2*^+^/*Nkx2-5*^+^/*Hcn4*^+^/*Scn5α*^+^ SAN peripheral cells possess electrophysiological property intermediate of the *Nkx2-5*^−^/*Shox2*^+^/*Hcn4*^+^/*Scn5α*^−^ SAN head and the *Shox2*^−^/*Nkx2-5*^+^/*Hcn4*^−^/*Scn5α*^+^ atrial cells. Such property enables the *Shox2*^+^/*Nkx2-5*^+^ SAN tail/peripheral domain to play a unique role in the S-A conduction in addition to its impulse generation capability. Consistent with this model are: (1) *Shox2*^Nkx2-5Cre^ mice exhibit sick sinus syndrome associated with a virtual absence of the SAN tail/peripheral domain [9]; (2) mice lacking *Tbx18* in the heart exhibit normal sinus rhythm regardless the lack of the SAN head [8]; and (3) a computational modeling predicts that the peripheral SAN cells possess higher maximum depolarization slope and higher AP amplitude than the SAN head cells to potentially function as a signal amplifier [59].

## 4. Genome-Wide Studies on Transcription Regulatory Networks and Chromatin Landscape in the Pro-Pacemaker Cells

It has been established that the SAN and pro-pacemaker cells in the venous pole possess a unique set of cellular properties and genetic features. This notion has been recently elaborated by transcriptome studies that demonstrate the possession of a distinct transcriptome profile by the SAN cells as compared with that in working myocardial cells [10]. It was further demonstrated that such unique SAN cell profile is at least in part maintained by the expression of *Isl1*, as deletion of *Isl1* by *Hcn4-CreER*^T2^ led to in the SAN an upregulation of working myocardial genes such as *Gja5* and *Gja1*, and a downregulation of pacemaker genes including *Tbx3*, *Shox2* and *Hcn4.* As abovementioned, *Shox2* and *Tbx3* are also responsible for the correct expression level of some of these genes in the SAN including *Isl1* itself. Such correlation suggests an epistatic link between *Shox2*, *Tbx3* and *Isl1* in SAN development. These genetic interactions, if tested by compounded allelic serial mutations of *Shox2*, *Tbx3*, and *Isl1*, will provide instrumental insights for a better understanding of the genetic cascades governing the unique pacemaker properties for the SAN and other pacemaker cells in the venous pole.

**Figure 4:**
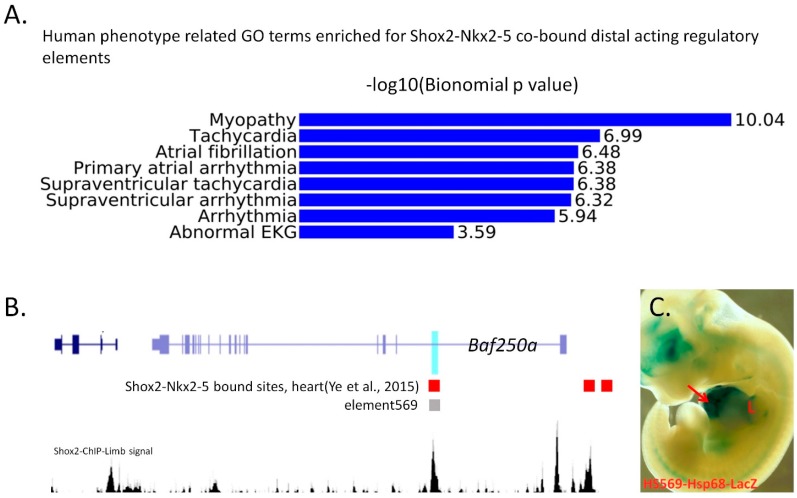
(**A**) Gene-ontology analysis reveals highly enriched atrial fibrillation related function in the Shox2-Nkx2-5 co-bound distal acting regulatory elements. (**B**) Signal track of Shox2-binding in the developing limb at E12.5 in relation to HS569, an enhancer of *Baf250a*, and Shox2-Nkx2-5 co-bound sites in the embryonic heart. (**C**) An E11.5 transgenic embryo carrying a reporter construct driven by Shox2-bound distal acting regulatory element (HS569) shows *LacZ* expression in the heart (arrow) and limb (L).

Furthermore, by ChIP-Seq, the molecular mechanisms involved in maintaining the particular properties of the pacemaker cells are being unveiled. Earlier studies of Tbx3 ChIP-Seq on adult heart using a *Tbx3* gain-of-function allele revealed a mechanism by which Tbx3 directly competes with Tbx5 to repress the working myocardial cell fate [58]. Similarly, a recent ChIP-Seq study on E12.5 mouse embryonic hearts unraveled an extensive genome-wide co-occupation of Shox2 and Nkx2-5, supporting the *Shox2*-*Nkx2-5* antagonistic mechanism by which Shox2 inhibits the transcription output of Nkx2-5 [9]. These lines of evidence together suggest the importance of transcription repressors in maintaining the pacemaker cell fate in a primitive state. Recent studies have shown that distal acting regulatory elements often reveal crucial information on the lineage specific chromatin landscape and reflect lineage specific transcription events as well as that transcription factor co-occupancy can be used to predict functional enhancers [60]. Our recent in-depth analyses of our published and unpublished ChIP-Seq data further identified co-occupation of Shox2 and Nkx2-5 on distal enhancers of a set of genes. Gene ontology (OG) analysis indicates that A-Fib related biological terms are highly enriched in the Shox2/Nkx2-5 co-occupied sites (Figure 4), suggesting that the co-occupation of Shox2 and Nkx2-5 can serve as a useful criterion for identifying A-Fib related *cis*-regulatory elements.

Interestingly, many of these elements discovered in the embryonic heart are also occupied by Shox2 in other developmental contexts. For example, a co-occupied site by Shox2 and Nkx2-5 in the intron-1 of *Arid1a* (*Baf250a*) in the embryonic heart [9] is also bound by Shox2 in the developing limb, suggesting that this site is accessible in both the developing heart and limb. Indeed, the orthologous region of this site in humans (HS569) [61] was characterized to have enhancer activity in the developing heart and limb in mice (Figure 4B,C). Furthermore, integrative interrogation of the distal regulatory elements identified by Islet1 ChIP-Seq in the postnatal SAN and that by Shox2 ChIP-Seq on the developing limb also unveils that a significant proportion of distal regulatory elements (~1/3) occupied by Islet1 is also bound by Shox2, indicating these regulatory elements are utilized in the development of both heart and limb [62]. Notably, a predominant proportion of these chromatin domains is not in an accessible configuration in the Encode heart DNasesHS dataset generated from working myocardium cells [63], suggesting that the pacemaker cells adopt *cis*-regulatory elements and associated transcription regulatory machinery that are also functioning in other developmental contexts to acquire the cellular phenotype distinct from working myocardial cells. Thus, integrative analysis of transcription factor binding profiles in other developmental context will aid for unraveling the transcriptional machineries operating in the developing SAN and pro-pacemaking cells in the venous pole.

Recent advance and deepened understanding on regulatory chromatin landscape point towards the possibility that based on the accessibility or particular histone modification on tissue/lineage specific *cis*-regulatory elements, the developmental competence of cells toward the lineage of desire can be predicted. This possibility was nicely exemplified by the studies on human embryonic stem cell derived pancreatic beta cells [64]. It would be important and interesting to explore the features of chromatin landscape in pacemaker cells that are responsible for the specific pacemaker properties including the distinctive sets of gene expression profiles. Such studies on pacemaker cells will similarly benefit the generation of biological pacemakers by providing readouts for the developmental and differentiation competence of precursors toward pacemaker cells. However, technically, it will be very difficult to profile the chromatin landscapes of pacemaker cells directly by DNase-HS, FAIRE-Seq or by ChIP-Seq on various histone modification markers that are predictive of active enhancer/promoter activity. This is due to the relatively scarce SAN cells and that the pro-pacemaker cells in the venous pole are patched with surrounding atrial myocardial cells, precluding accurate isolation of sufficient amount of pacemaker cells in an undisturbed *in vivo* state. Currently, most of the SAN markers, such as Tbx3 and Hcn4, also label other conduction components, including the AVN. A *Shox2* knock-in allele that expresses the *Shox2a* isoform and *DsRed* (*Shox2*^HA-DsRed^) and allows live imaging of *Shox2* expression in the venous pole and the developing SAN [9] would offer a unique tool for FACS isolation of venous pole pacemaker cells. In conjunction with open chromatin sequencing techniques that were designed for rare population of cells such as ATAC-Seq [65], it would be quite possible to characterize the accessible chromatin landscape of pacemaker cells comprehensively. Nevertheless, current ChIP-Seq studies on transcription factors closely related to pacemaker development in the venous pole provide a glimpse into the chromatin landscape of pacemaker cells.

## 5. Conclusions

**Figure 5:**
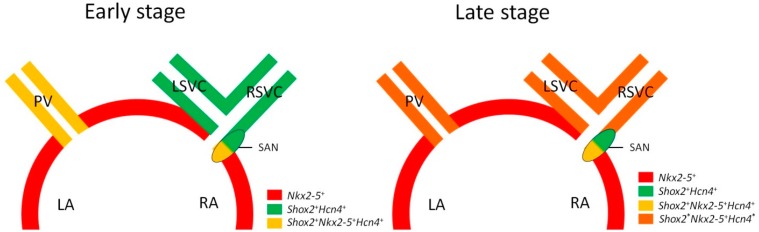
A schematic illustration summarizing the fate decision of cells in the developing venous pole. In the early developing venous pole, *Shox2* is expressed in the SAN head domain as well as the myocardium of the LSVC and RSVC, which are all positive for *Hcn4*. In addition, the SAN tail/peripheral domain and the PV myocardium are positive for *Shox2*, *Nkx2-5*, and *Hcn4*. At the late developmental stage and adult, the myocardium of the PV, LSVC, and RSVC becomes matured and largely adopts working myocardial phenotype, with potential to reacquire pacemaking activities.

In this review we summarize the previous studies in the perspective of SAN and pro-pacemaking venous pole development (Figure 5). In light of these comprehensive sets of studies, we propose that a delayed maturation towards working myocardium phenotype underpins the pacemaker properties of the *Shox2*^+^ pro-pacemaker cells in the developing SV, PV and SAN. Moreover, a converged repressive transcription output of *Isl1*, *Tbx3*, *Tbx18*, and *Shox2* for working myocardial program appears essential for the solidification of a morphologically distinct SAN. Additional transcription activators that are expressed in the venous pole, such as *Gata4*/6 and CoupTFII, may play crucial role to enforce the *in vivo* developmental program in the SAN.
